# Cathepsin V drives lung cancer progression by shaping the immunosuppressive environment and adhesion molecules cleavage

**DOI:** 10.18632/aging.205278

**Published:** 2023-12-08

**Authors:** Lifei Zhu, Qi Zeng, Jinxiang Wang, Fan Deng, Shi Jin

**Affiliations:** 1 Department of Cell Biology, School of Basic Medical Sciences, Southern Medical University, Guangzhou 510515, China; 2 Department of Dermatology, Guangdong Provincial People’s Hospital, Guangdong Academy of Medical Sciences, Guangzhou 510080, China; 3 Department of Oncology, The Fifth Affiliated Hospital of Sun Yat-sen University, Zhuhai 519000, China; 4 Guangdong Provincial Key Laboratory of Digestive Cancer Research, Digestive Diseases Center, Precision Medicine Center, Sun Yat-Sen University, Shenzhen 518107, China; 5 Scientific Research Center, The Seventh Affiliated Hospital, Sun Yat-Sen University, Shenzhen 518107, China; 6 National Cancer Center/National Clinical Research Center for Cancer/Cancer Hospital and Shenzhen Hospital, Chinese Academy of Medical Sciences and Peking Union Medical College, Shenzhen 518116, China

**Keywords:** CTSV, adhesion, fibronectin, E-cadherin, N-cadherin

## Abstract

Cathepsin V (CTSV) is a cysteine cathepsin protease that plays a crucial role in extracellular matrix degradation. CTSV is correlated with poor prognosis in various cancers, but the underlying mechanism remains elusive. Here, we observed that CSTV is upregulated in lung cancer and is a poor prognosis factor for lung cancer. CTSV acts as a driver in the metastasis of lung cancer both *in vitro* and *in vivo*. CTSV promotes lung cancer metastasis by downregulating adhesion molecules, including fibronectin, E-cadherin, and N-cadherin. Our data revealed that CTSV functions by mediating the fragmentation of fibronectin, E-cadherin, and N-cadherin in cleavage, remodeling the extracellular matrix (ECM). The rationally designed antibody targeting CTSV blocks its cleaving ability towards fibronectin, E-cadherin, and N-cadherin, suppressing migration and invasion. Furthermore, we found that CTSV expression is negatively correlated with immune cell infiltration and immune scores and inhibits T cell activity. Targeting CTSV with specific antibodies effectively suppressed lung cancer metastasis in a mouse model. Our study demonstrates the critical role of CTSV in the immunity and metastasis of lung cancer, suggesting that the CTSV-targeting approach is a promising strategy for lung cancer.

## INTRODUCTION

Lysosomal peptidases are known as cysteine cathepsins. The cysteine cathepsin family is comprised of 11 members (CTSB, CTSF, CTSC, CTSK, CTSH, CTSO, CTSL, CTSV, CTSS, CTSW and CTSX) [1]. Cysteine proteases usually act as endopeptidases, which cleave the peptide bonds with substrate proteins [2]. As reported, CTSB holds the property as a carboxypeptidase and CTSH as an aminopeptidase. CTSC functions as a dipeptidyl aminopeptidase, and CTSZ is a carboxymonopeptidase [3, 4]. Activated immune and stromal cells discharged cathepsin proteases into the extracellular space [5, 6], whereas the cysteine cathepsins’ molecular mechanisms in extracellular space remain unclear and an open question.

Cysteine cathepsins are involved in both the general breakdown of proteins in cells and the regulation of some specific physiological processes [7–9]. When protease/anti-protease equilibrium is out of balance, this active system of controlled protein hydrolysis may be shut off, linked to various diseases, including osteoporosis, atherosclerosis, and cancer [2, 10]. Dysregulated transcription, localization, compartmentalization, activation of the enzyme progenitor, or an imbalance in endogenous inhibitor activity are the causes of this elevated activity [10]. Additionally, recent developments in proteomics and degradation have made it possible to identify several new substrates for cysteine proteases, furthering our comprehension of the protease family’s biological functions and biomarker applications [11, 12].

High cathepsin X level in serum was co-related with poor overall survival in colorectal (CRC) [13, 14], and cathepsin W was proved to be involved in the immunity and metastasis of CRC [15]. A higher serum level of cathepsin L in malignant ovarian cancer is a prognostic marker correlated with poorer survival [16]. A study of more than 2,000 individuals showed that elevated serum CTSS levels are associated with increased mortality in older adults [17]. Cathepsin B is also transported to the extracellular surface of the plasma membrane, where it interacts with membrane-bound proteins annexin 2 in the pit [18]. Cathepsin C promotes lung colonization of breast cancer by activating neutrophil infiltration and NET formation [19]. Inhibiting cathepsin C suppresses autophagy and reduces the growth of colon cancer [20]. Cathepsin expression is regulated according to cell type in these different cell populations, and their promoting or suppressing functions in tumors may vary in similar ways [8]. Elevated expression of CTSV is associated with many malignant tumors, such as breast cancer, squamous cell cancer, and colon-rectal cancer. The reduced severity of arthritis in cathepsin L-deficient mice is due to damage to the T helper cell region [21]. By boosting NF-B activity, cathepsin V speeds up the development of bladder cancer and suppresses the production of the GATA3 protein in luminal A breast cancer [22]. HMGB1 regulated homocysteine-induced endothelial cell pyroptosis by CTSV-dependent pathway [23]. N-glycosylation of CTSV is necessary for its transportation and secretion [24]. A recent study has reported that CTSV in serum is a prognosis biomarker in lung cancer [25]. After that, exploring the molecular mechanisms of how CTSV drives lung cancer tumorigenesis is a promising study.

According to this study, CTSV increased lung cancer metastasis by lowering fibronectin, E-cadherin, and N-cadherin adhesion molecules. By modifying fibronectin, E-cadherin, and N-cadherin cleavage and fragmentation, we discovered that CTSV promoted tumor spread. We created an antibody that specifically targets CTSV and successfully blocks its ability to cleave fibronectin, E-cadherin, and N-cadherin, which reduces tumor cell invasion and migration. Furthermore, CTSV reduces T cell function *in vitro*, and CTSV antibody inhibition significantly reduces lung cancer metastasis.

## MATERIALS AND METHODS

### Raw data collection

The RNA sequencing lung cancer data and the clinical outcome information were collected from the TCGA database (http://www.tcga.org/).

### Analysis of the TIMER database

We investigated the relationship between immune cell infiltration level and CTSV expression using the online tool TIMER (https://cistrome.shinyapps.io/timer/) and utilized it to define the immunological environment in lung cancer in various levels of CTSV gene copying model. Statistics were defined as significant at *P* < 0.05.

### Cell culture and transfection

In this study, all human lung cancer cell lines A549, NCI-H1975, NCI-H292 cells, and the Human Embryonic Kidney 293T cells were purchased from the American Type Culture Collection (ATCC) and were cultured according to the instructions. HEK293T, HEK293T, A-549, and NCI-H292 were cultured using DMEM medium (Gibco, USA) supplemented with 10% fetal bovine serum (Excell), NCI-H1975 cells were cultured with RPMI-1640 (Gibco, USA) supplemented with 10% fetal bovine serum. All cells were maintained at 37°C and 5% CO_2_ incubator. To establish stable tumor cells, lentiviruses carrying shRNAs (2 μg DNA) were used to infect the cells in RPMI-1640 (Gibco) supplemented with 10% fetal bovine serum, followed by the standard procedure for Lipofectamine 3000 Transfection Reagent (Thermo Fisher, NY, USA). Finally, the stable cells were selected by 0.5 μg/ml puromycin. For HA-tagged CTSV plasmid construction, 0.5 and 1 μg CTSV plasmid were used and transfected as previously. The knockdown sequence of CTSV is shown as follows: shCTSV#1: TTCCAAAATTTGACCAAAATTTG, shCTSV#3: TCCAAAATTTGACCAAAATTTGG.

### Migration and invasion assays

We used 200 μl of serum-free DMEM to seed (1 × 10^5^) A549, NCI-H1975, and NCI-H292 cells in the 24-well Boyden chamber (BD Biosciences, USA) with an 8 m pore size. The bottom compartment was filled with a solution containing 20% fetal bovine serum in DMEM medium. To perform the migration assay, we cultured the cancer cells in the membrane devoid of an extracellular matrix covering for 6 h. We seeded the cancer cells in the membrane treated with 50 μl of 1:8 diluted Matrigel (BD Biosciences, USA) for the invasion assay. The Boyden chamber was then incubated for roughly 10 h at 37°C with 5% CO_2_. The cells that had passed through the bottom chamber’s insert had been preserved, stained, and then viewed under a microscope.

### Adhesion testing

Using culture plates that had been prepared with 10% BSA to prevent nonspecific binding protein and fibronectin, cell adhesion to fibronectin (10 μg/ml, BestBio) was measured. Cells from the A549 and NCI-H1993 strains were suspended in serum-free media at 37°C and 5% CO_2_ for 30 min. The cells were gently washed three times in PBS to remove any adhering cells. Crystal violet (0.005%, Sigma-Aldrich, USA) was used to stain the adherent cells, which were counted under a microscope. After the incubation, the associated number of cells was expressed as a percentage of the total number of cells.

### Co-culture assay and cellular apoptosis detection

Seeded A549 and NCI-H1975 cells (1 × 10^6^) into 6-well plates in 2 ml of media and incubated for 24 h. After being stimulated for 24 h with 50 ng/L PMA and 1 μg/ml Ionomycin, peripheral blood mononuclear cells (PBMCs) from healthy individuals were introduced to lung cancer cells at a ratio of 2:1. Cellular apoptosis of A549 and NCI-H1975 cells were determined by Caspase-Glo 3/7 assay kits (Promega, USA), the activity of Caspase 3 and Caspase 7 were measured following the manufacturer’s protocol.

### Western blotting

After removing the cells from the incubator and discarding the media, pre-cold PBS was used to wash the cells. Placing the cells on ice and adding the RIPA lysis solution with cocktail protease and phosphatase inhibitors (Sigma-Aldrich, USA) will cause the cells to lyse. For 30 min, cells were lysed on ice. Utilizing a cell scraper, centrifuge the cells for 20 min at 4°C at 16128 RCF/g. On the 10% SDS-Page gel, 20 μg of total protein was added and separated. The obtained protein was then deposited onto 12 × 9 cm^2^ PVDF membranes (Millipore), which were then blocked for an h at room temperature in 5% non-fat milk. Then, 12–16 h at 4°C in primary antibody incubation. A horseradish peroxidase-conjugated secondary antibody was added, and a MiniChmei Chemiluminescence imager was used to detect the results.

### Immunoprecipitation

After removing the cells from the incubator and discarding the media, pre-cold PBS was used to wash the cells. After placing the cells on ice, add RIPA lysis solution (Sigma-Aldrich, USA), which contains a cocktail of protease and phosphatase inhibitors. For 30 min, cells were lysed on ice. Scrape off the cells with a cell scraper centrifuged at 16128 RCF/g for 20 min. Took 30 μL supernatant as Input group, add 5 × loading buffer, 100°C for 10 min. Added agarose beads to the supernatant (if agarose beads without antibodies, both antibodies and Protein G-Agarose should be used, and IgG antibodies and agarose beads are added to the control group), incubated for 6 h at 4°C on a vertical shaker for 10–12 rpm turnover. Finally, the samples were centrifuged at 4°C and 16128 RCF/g for 1 min, the supernatant was discarded, and agarose beads were washed with RIPA 5 times. Added 5×loading buffer, 100°C for 10 min, and the mixture was directly used for Western Blot detection.

### Immunofluorescence

For immunofluorescence, cells were sown in 8-well chamber slides at a confluence of around 50% and fixed with 0.2% Triton X-100 in PBS for 20 min. Cells were first blocked in PBS containing 3% BSA for 1 h at room temperature after being washed thrice in PBS. Blocking buffer was used to dilute the primary antibodies (1:200) before they were incubated at 4°C overnight. Cells were treated with Alexa Fluor-conjugated secondary antibodies (Invitrogen, USA) for 1 h at room temperature after being diluted in PBS (1:1000). After that, the nuclei were counterstained with DAPI (1:10000).

### Protein mass spectrometry (MS) analysis

A549 and NCI-H1975 cells were transfected with Flag-tagged CTSV plasmids. NaF (15 mmol/L), -glycerophosphate (60 mmol/L), pepstatin A (1 mg/ml), and aprotinin (1 mg/ml) were added to a buffer to lyse the cells. The Cell lysates were centrifuged for 10 min at 15,000 g 4°C to remove debris. The supernatant was incubated with Flag-conjugated beads at 4°C for 8 h. Beads were washed 5 times using the buffer we previously described, 100°C for 10 min. Samples were then analyzed by SDS-PAGE and immunoblotting and subjected to mass spectrometry analysis to identify individual proteins.

### Antibodies

Antibodies against CTSV (WB: Abcam, Cat#ab166894), Fibronectin (Abcam, Cat#ab268020), E-cadherin (Cell Signaling Technology, Cat#3195S), N-cadherin (Cell Signaling Technology, Cat#13116S), HA-Tag (Cell Signaling Technology, Cat#3724S), Cleaved-Caspase 3 (Cell Signaling Technology, Cat#9654S), Cleaved-Caspase 7 (Cell Signaling Technology, Cat#8438S), Tubulin (Genetex, Cat#GTX112141), GAPDH (Genetex, Cat#GTX100118) were purchased from the indicated companies.

### Xenograft lung cancer model and CTSV antibody generation

Animal studies were conducted strictly in line with the “Principles for the Utilization and Care of Vertebrate Animals” and the “Guide for the Care and Use of Laboratory Animals,” as approved by the Animal Research Committee of the Sun Yat-sen University Cancer CenterStable luciferase-expressing A549 cells (A549-luci) and cells derived from A549-luci were created. In the animal tests, male BALB/c nude mice aged four to six weeks were employed. Following the injection of the vain tail, the mice were split into two groups. Used 0.9% NaCl and CTSV antibody to treat the appropriate mice on d 24, 30, and 36. The mice bearing A549 tumor cells stably expressed luciferase were monitored for metastasis using an IVIS Lumina Imaging System.

The amino acid sequence for the CTSV antibody is shown as follows: “PYVAVDEICKYRPENSVANDTGFTVVAPGKEKALMKAVATVGPISVAMDAGHSSFQFYKSGIYFEPDCSSKNLDHGVLVVGYGFEGANSNNSKYWLVKNSWGPEWGS”. Animals are immunized with the antigen protein specific to the human CTSV to produce monoclonal antibodies.

### Statistical analysis

The data presented as mean standard deviation (SD) were gathered from two or three studies. To analyze the data, we utilized the unpaired two-tailed Student’s *t*-test module, where ns denotes no statistical significance, ^*^P-value < 0.05, ^**^P-value < 0.01, ^***^P-value < 0.001, and ^****^P-value < 0.0001. The graphs were created with the GraphPad Prism program. Biorender (https://www.biorender.com/) created the Graphical Abstract. Three separate experiments were used to get representative immunofluorescence data. Statistics were defined as significant at *P* < 0.05.

### Availability of data and materials

The data used or analyzed in this study are available from the corresponding author on reasonable request.

## RESULTS

### CTSV is overexpressed in lung cancer and correlated with poor survival

**Figure 1:**
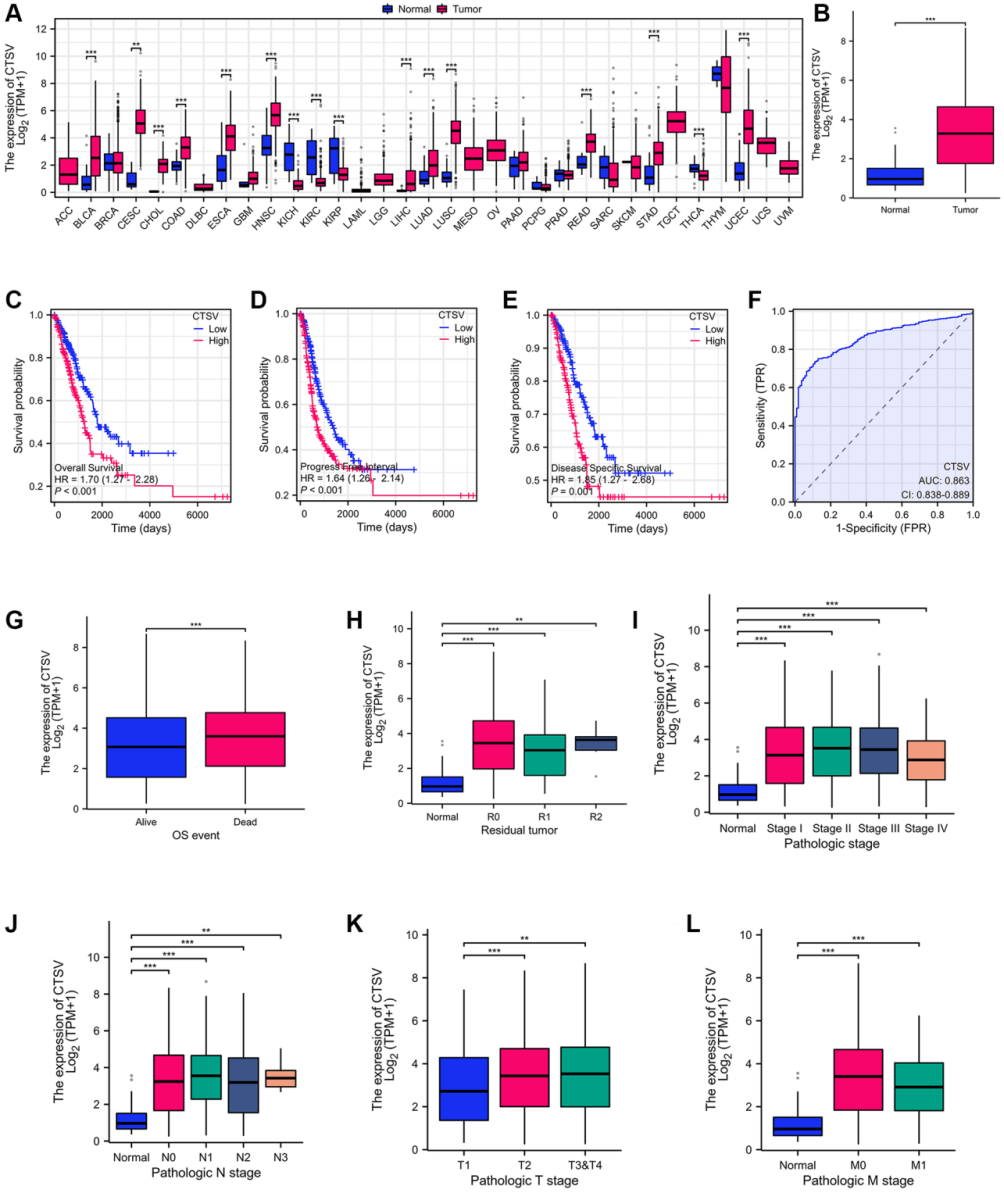
**Identification of CTSV as the driver for malignant progression of lung cancer.** (**A**) CTSV expression analysis in pan-cancer using tumor tissues paired with normal tissues from the TCGA database. (**B**) CTSV expression analysis of lung cancer using lung cancer and normal samples from the TCGA database. (**C**–**E**) Kaplan-Meier survival curves based on CTSV expression in the lung cancer samples. (**F**) ROC analysis of lung cancer based on CTSV expression. (**G**, **H**) The expression of CTSV was analyzed based on OS events and residual tumor. (**I**–**L**) Relationship between CTSV expression and clinical outcomes including (**I**) Pathologic stage, (**J**) N stage, (**K**) T stage, (**L**) M stage. ^*^P < 0.05, ^**^P < 0.01, ^***^P < 0.001.

With the aid of the TCGA database, we carried out a pan-cancer analysis to look into CTSV expression in tumors. According to the findings, lung cancer is one type of tumor with elevated CTSV expression (Figure 1A, 1B). Next, we used prognosis data from the TCGA database to determine if CTSV is associated with clinical outcomes. The data showed that CTSV is positively correlated with overall survival (OS), progression-free interval (PFI), and disease-specific survival (DSS) (Figure 1C–1E). The area under the curve (AUC) value for CTSV is 0.863 (95% CI: 0.838–0.889) (Figure 1F), which indicates that it may be a viable biomarker for people with lung cancer. Moreover, multivariate logistic regression analysis revealed that CTSV expression was associated with prognostic factors. Elevated expression of CTSV was correlated with OS event (Figure 1G), residual tumor (Figure 1H), Pathologic stage (Figure 1I), N stage (Figure 1J), T stage (Figure 1K), and M stage (Figure 1L). Cellular cancers with high levels of CTSV had poor survival rates, suggesting that CTSV may be a useful prognostic biomarker for lung cancer.

### CTSV drives the metastasis of lung cancer

**Figure 2:**
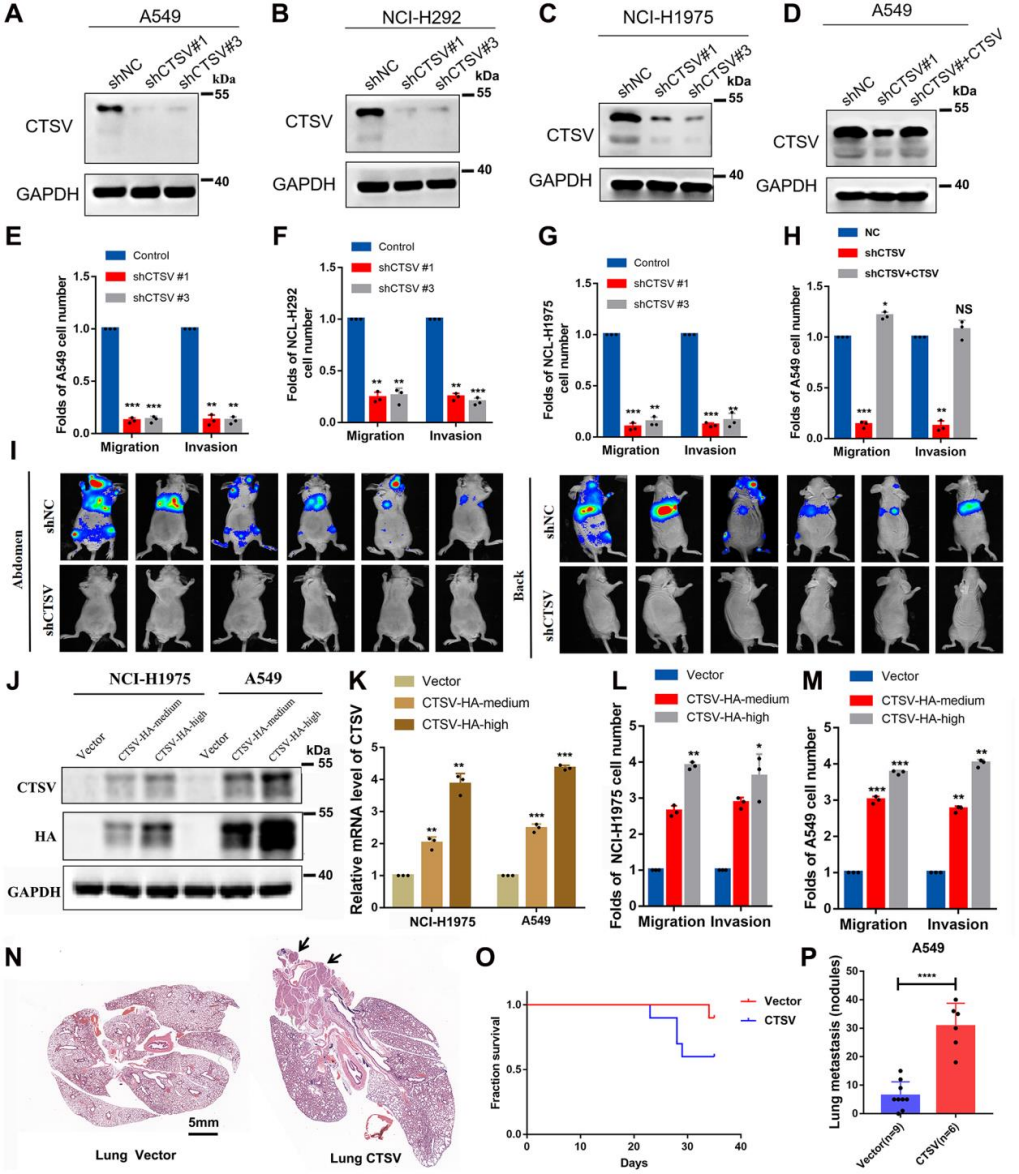
**CTSV contributes to the metastasis of lung cancer cells both *in vitro* and *in vivo*.** (**A**–**C**) A549, NCI-H292, and NCI-H1975, lung cancer cells transfected with the indicated negative control plasmids, shCTSV#1 or shCTSV#3, for 48 h were analyzed by Western blotting. The results were repeated for three biologically independent experiments. (**D**) The indicated stable cells transfected with shCTSV#1 and HA-CTSV for 48 h were subjected to Western blotting. The grouping of blots cropped from different parts of the same gel. (**E**–**H**) The indicated stable cells were analyzed by cell migration and invasion assays. The results are the mean ± SD of three biologically independent experiments. ^*^P < 0.05, ^**^P < 0.01, ^***^P < 0.001 were analyzed by using the student’s *t*-test. NS represents no significance. (**I**) Bioluminescent imaging analysis of mice. Representative images are shown (*n* = 6 mice per group). The tumors were isolated at the end of the experiments. (**J**–**M**) CTSV-HA-medium or CTSV-HA-high NCI-H1975 and A549 cells were transiently transfected with sgRNAs as indicated and then analyzed by Western blotting, qRT-PCR, cell migration, and invasion assays. The results showed that with the increase in CTSV expression (2–5 times the normal value), the promoting effect of CTSV on migration and invasion also improved. The grouping of blots cropped from different parts of the same gel. (**N**–**P**) H&E staining of lungs from representative tumor-bearing nude mice. Scale bars, 5 mm. Kaplan-Meier survival curves of the nude mice in the vector and CTSV-HA groups. Quantification of lung nodules. Data are shown as the mean ± SD and *P*-values; two-tailed Student’s *t*-tests were used to analyze the data. ^****^P-value < 0.0001. Full-length blots are shown in Supplementary Figure 1.

We used two pairs of short hairpin RNAs (shRNAs) to suppress CTSV expression in three lung cancer cell lines, A549, NCI-H292, and NCI-H1975, to determine the biological role of CTSV in the progression of lung cancer (Figure 2A–2C) In A549, NCI-H292 and NCI-H1975 cells, endogenous CTSV depletion with shRNAs had a strikingly detrimental effect on migration and invasion (Figure 2E–2G). This effect was entirely reversed by the reintroduction of CTSV (Figure 2D, 2H). We carried out lung cancer xenograft mice model experiments to examine the *in vivo* functional impact of CTSV on carcinogenesis. Notably, in an *in vivo* lung cancer metastasis model using the stable cell line A549-luciferase, CTSV knockdown significantly reduced lung and distant metastases (Figure 2I). In the following step, we successfully transfected lung cancer cell lines and stably overexpressed CTSV to varying degrees at the protein and mRNA levels (2 to 4 times greater than usual). To imitate the deregulated stages of pathologic processes, we overexpressed CTSV using various concentration gradient lentiviruses (middle with 2–3 times and high with 4–6 times) (Figure 2J, 2K). NCI-H1975 and A549 cell movement and invasion were greatly aided by CTSV overexpression (Figure 2L, 2M).

Additionally, we used the stable cell line A549 to create an *in vivo* tail vein model of lung cancer metastasis. Overexpression of CTSV significantly accelerated lung metastases, as shown by an increase in the number of metastatic nodules in the lungs, and shortened the survival time of nude mice (Figure 2N–2P). In conclusion, CTSV knockdown inhibited lung cancer’s ability to develop malignantly, and its detrimental effects on cancer metastasis led to its oncogenic activity.

### CTSV drives metastasis of lung cancer by downregulating adhesion molecules

**Figure 3:**
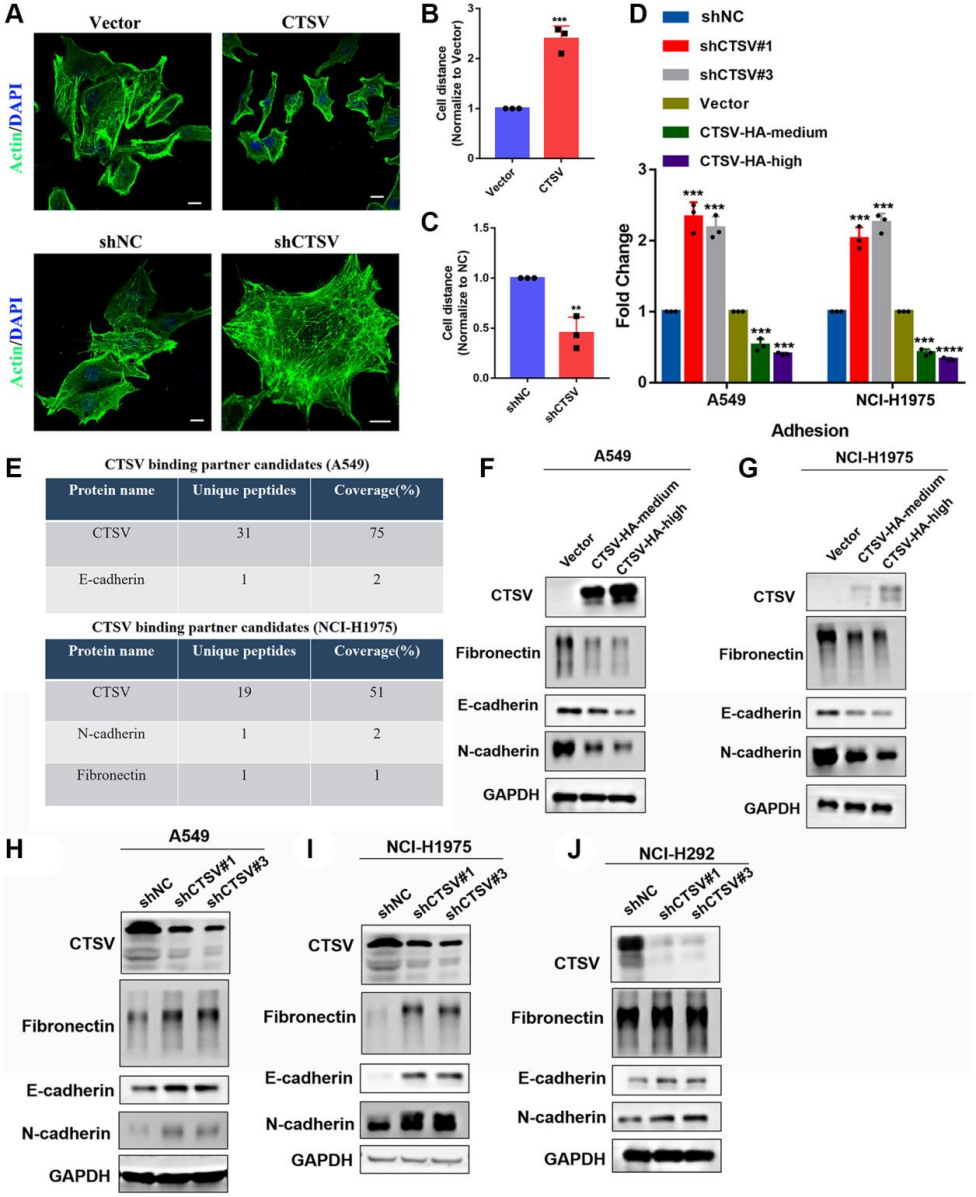
**CTSV promotes lung cancer metastasis by cleaving off adhesion proteins.** (**A**–**C**) Immunofluorescence analysis was performed using an anti-actin antibody in the indicated stable cells. Scale bar, 50 μm. (**D**) The indicated A549/NCI-H1975 cells were analyzed by adhesion assay. (**E**) Protein mass spectrometry analysis of CTSV binding partner candidates in the indicated stable cells. (**F**, **G**) A549 and NCI-H1975 cells expressing CTSV-HA-medium and CTSV-HA-high were analyzed by Western blotting using anti-fibronectin, anti-E-cadherin and anti-N-cadherin antibodies. (**H**–**J**) A549, NCI-H1975, and NCI-H292 cells transfected with negative control plasmids, shCTSV#1 or shCTSV#3 for 48 h were analyzed by Western blotting using anti-fibronectin, anti-E-cadherin and anti-N-cadherin antibodies. The grouping of blots cropped from different parts of the different gels. Data are shown as the mean ± SD and repeated for three biologically independent experiments. *P*-values are shown; two-tailed Student’s *t*-test. Abbreviation: NC: negative control shRNA. Vector, vector-only control. The representative data are repeated for three biologically independent experiments. Student’s *t*-tests were used for data analysis; ^*^P < 0.05, ^**^P-value < 0.01, ^***^P-value < 0.001 and ^****^P-value < 0.0001.

We carried out immunofluorescence experiments in CTSV-overexpressing and CTSV knockdown A549 cells to learn more about the role of CTSV in lung cancer. We discovered that CTSV knockdown had the opposite effects of CTSV overexpression, increasing cell-cell distance while decreasing cell-to-cell adhesion (Figure 3A–3C). Additionally, our adhesion assay revealed that CTSV knockdown boosted the level of cell-to-cell adhesion while CTSV overexpression considerably lowered it (Figure 3D). Observations from the past suggest that CTSV will alter the ECM environment by interacting with specific substrates. To do this, we used protein mass spectrometry on both NCI-H1975 and A549 cells, and the results showed that CTSV interacts with the crucial molecules fibronectin, E-cadherin, and N-cadherin for metastasis (Figure 3E). Increased cathepsin activity is essential for breaking down cell-cell adhesion molecules, which promotes metastasis and invasion [16, 26]. However, little is known about the underlying molecular mechanism of how CTSV promotes metastasis. We want to determine if CTSV controls these adhesion molecules that promote metastasis.

Fibronectin, E-cadherin, and N-cadherin protein levels were lowered in A549 and NCI-H1975 cells by stable exogenous CTSV expression (Figure 3F, 3G). According to the data above, fibronectin, E-cadherin, and N-cadherin protein levels were considerably elevated in A549, NCI-H1975, and NCI-H292 cells after CTSV was knocked down with shRNAs (Figure 3H–3J). However, fibronectin, E-cadherin, or N-cadherin were unaffected by CTSV expression at the mRNA level (Supplementary Figure 1), proving that CTSV altered the protein levels of fibronectin, E-cadherin, and N-cadherin.

**Figure 4:**
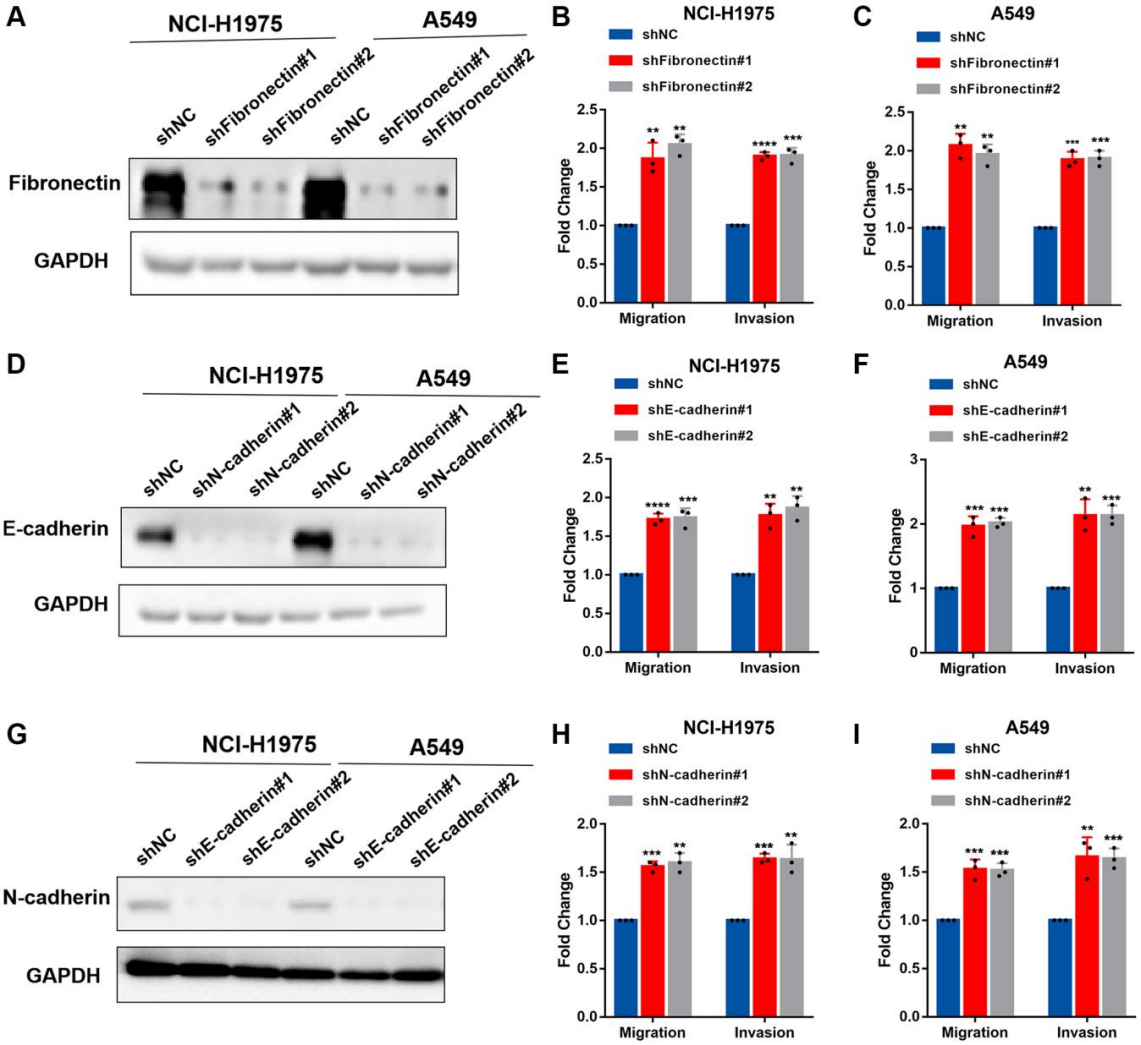
**Fibronectin, E-cadherin, and N-cadherin regulate cell adhesion, migration, and invasion.** (**A**–**C**) NCI-H1975 and A549 cells were transfected with shRNAs targeting fibronectin and then analyzed by Western blotting, migration and invasion assay. (**D**–**F**) NCI-H1975 and A549 cells were transfected with shRNAs targeting E-cadherin and then analyzed by Western blotting, migration and invasion assay. (**G**–**I**) NCI-H1975 and A549 cells were transfected with shRNAs targeting N-cadherin and then analyzed by Western blotting, migration and invasion assay. The grouping of blots cropped from different parts of the different gels. Data are shown as the mean ± SD and repeated for three biologically independent experiments. *P*-values are shown; two-tailed Student’s *t*-test. Abbreviation: NC: negative control shRNA. Vector, vector-only control. The representative data are repeated for three biologically independent experiments. Student’s *t*-tests were used for data analysis; ^*^P < 0.05, ^**^P-value < 0.01, ^***^P-value < 0.001 and ^****^P-value < 0.0001.

We knocked down the expression of fibronectin, E-cadherin, and N-cadherin and performed invasion and migration assays in NCI-H1975 and A549 cells to examine the biological function of fibronectin, E-cadherin, and N-cadherin concerning metastasis. According to the data, lung cancer cells’ capacity to invade and migrate was dramatically boosted when fibronectin, E-cadherin, and N-cadherin expression was knocked down (Figure 4A–4I). Overall, our findings indicated that CTSV downregulates adhesion molecules such as fibronectin, E-cadherin, and N-cadherin, which may suggest that CTSV is a potential therapeutic target for halting lung cancer spread.

### CTSV-mediated fragmentation of fibronectin, E-cadherin, and N-cadherin in cleavage

**Figure 5:**
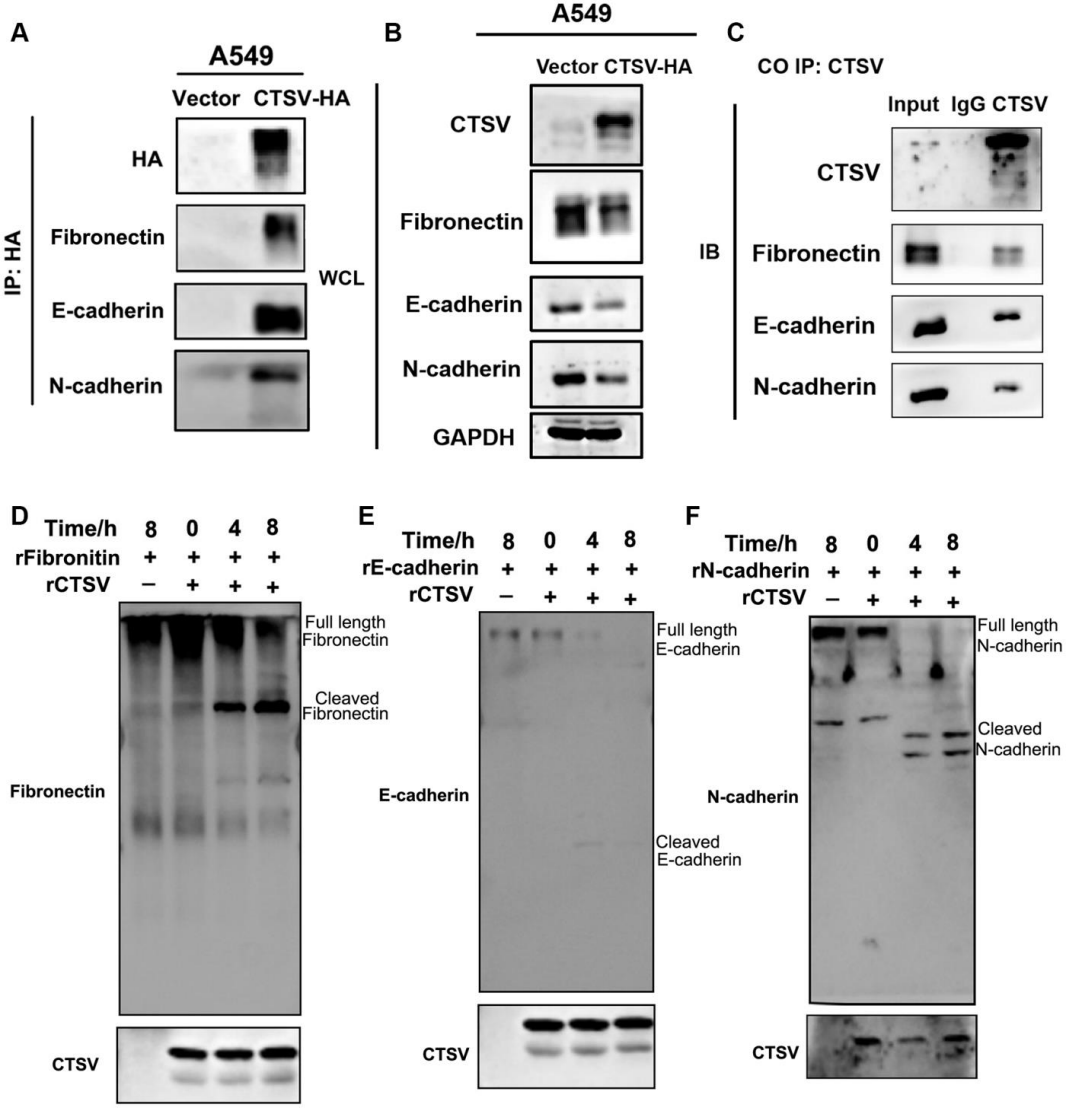
**CTSV cleaves off fibronectin, E-cadherin, and N-cadherin.** (**A**, **B**) Stable CTSV-HA A549 cells were subjected to coimmunoprecipitation assays using anti-fibronectin, anti-E-cadherin, and anti-N-cadherin antibodies, followed by Western blotting thrice. The grouping of blots cropped from different parts of the different gels. (**C**) Lysates of A549 cells were subjected to IP using anti-fibronectin, anti-E-cadherin, and anti-N-cadherin antibodies and control IgG, then detected by immunoblotting. (**D**–**F**) 1 μg recombinant human fibronectin, E-cadherin, and N-cadherin were incubated with 1 μg recombinant human CTSV at 37°C for the indicated periods. Fibronectin, E-cadherin, and N-cadherin fragments were detected using anti-fibronectin, anti-E-cadherin, and anti-N-cadherin antibodies. HA is shown as a control. The grouping of blots cropped from different parts of the same gels.

Therefore, proteases take a role in tumor cell invasion and metastasis by mediating protein degradation and extracellular matrix (ECM) remodeling by cleaving off the ECM to open intercellular connections [27]. According to studies, aberrant proteolytic activity may lead to illness problems [28]. Patients with metastasized lung cancer have been shown to have an overexpression of the proteolytic enzyme CTSV, which indicates a worse prognosis [25]. CTSV interacts with fibronectin, E-cadherin, and N-cadherin (exogenous) according to the coimmunoprecipitation experiment (Figure 5A, 5B). Additionally, endogenous fibronectin, E-cadherin, and N-cadherin could be pulled down after immunoprecipitation of CTSV using an anti-CTSV antibody (Figure 5C).

An anti-fibronectin antibody was used to detect a fibronectin fragment product after the two recombinant proteins, fibronectin and CTSV, were incubated (Figure 5D). Recombinant human E- and N-cadherin were treated with recombinant CTSV proteins, and similar fragments emerged (Figure 5E, 5F). These findings imply that CTSV cleaves fibronectin, E-cadherin, and N-cadherin to act as a pro-metastatic indication.

### CTSV blockade inhibits the migration and invasion of lung cancer cells

**Figure 6:**
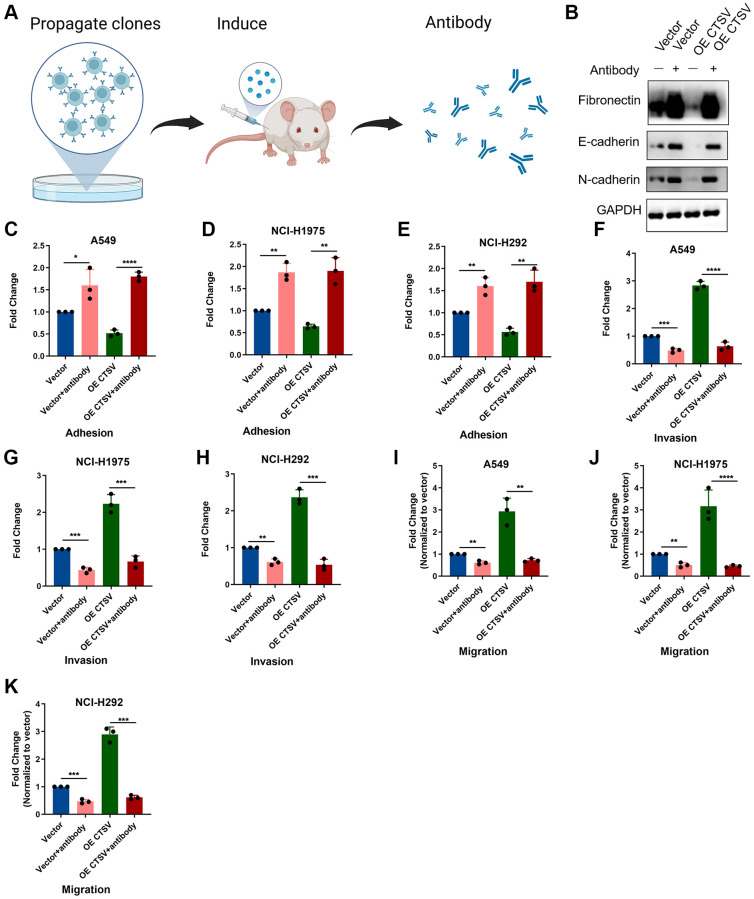
**CTSV antibody blockade inhibits adhesion, migration, and invasion in lung cancer cells.** (**A**) The schematic diagram for the experimental procedures of generating CTSV antibodies. (**B**) A549 cells treated with CTSV antibody were then subjected to immunoblotting assay using anti-fibronectin, anti-E-cadherin, and anti-N-cadherin antibodies. The grouping of blots cropped from different parts of the same gels. Full-length blots are shown in Supplementary Figure 1. (**C**–**E**) Adhesion assays were performed for the indicated stable A549, NCI-H1975, and NCI-292 cells pre-treated with CTSV antibody. (**F**–**K**) The indicated stable A549, NCI-H1975, and NCI-H292 cells were treated with CTSV antibody and examined by migration and invasion assay. The representative data are repeated for three biologically independent experiments. Student’s *t*-tests were used for data analysis; ^**^P-value < 0.01, ^***^P-value < 0.001, and ^****^P-value < 0.0001.

Recent biological advances have brought new strategies for blocking certain proteins by antibodies to improve anticancer efficacy. Here, we generated an antibody targeting CTSV to explore its role in clinical benefits (Figure 6A). We used CTSV antibodies on cells that were ectopically expressing CTSV to characterize the role of the antibody in cleaving fragmentation of fibronectin, E-cadherin, and N-cadherin. As shown in the western blotting data, CTSV blockade significantly increased the protein level of fibronectin, E-cadherin, and N-cadherin (Figure 6B). We then investigate how lung cancer cells’ abilities for adhesion, migration, and invasion are affected by CTSV inhibition. A549, NCI-H1975, and NCI-H292 cells’ ability to adhere to one another was enhanced by CTSV inhibition (Figure 6C–6E), while migration and invasion were prevented (Figure 6F–6L). These findings showed that lung cancer cells’ ability to migrate and invade was decreased by CTSV inhibition.

### CTSV expression is negatively associated with active tumor-infiltrating immune cells

**Figure 7:**
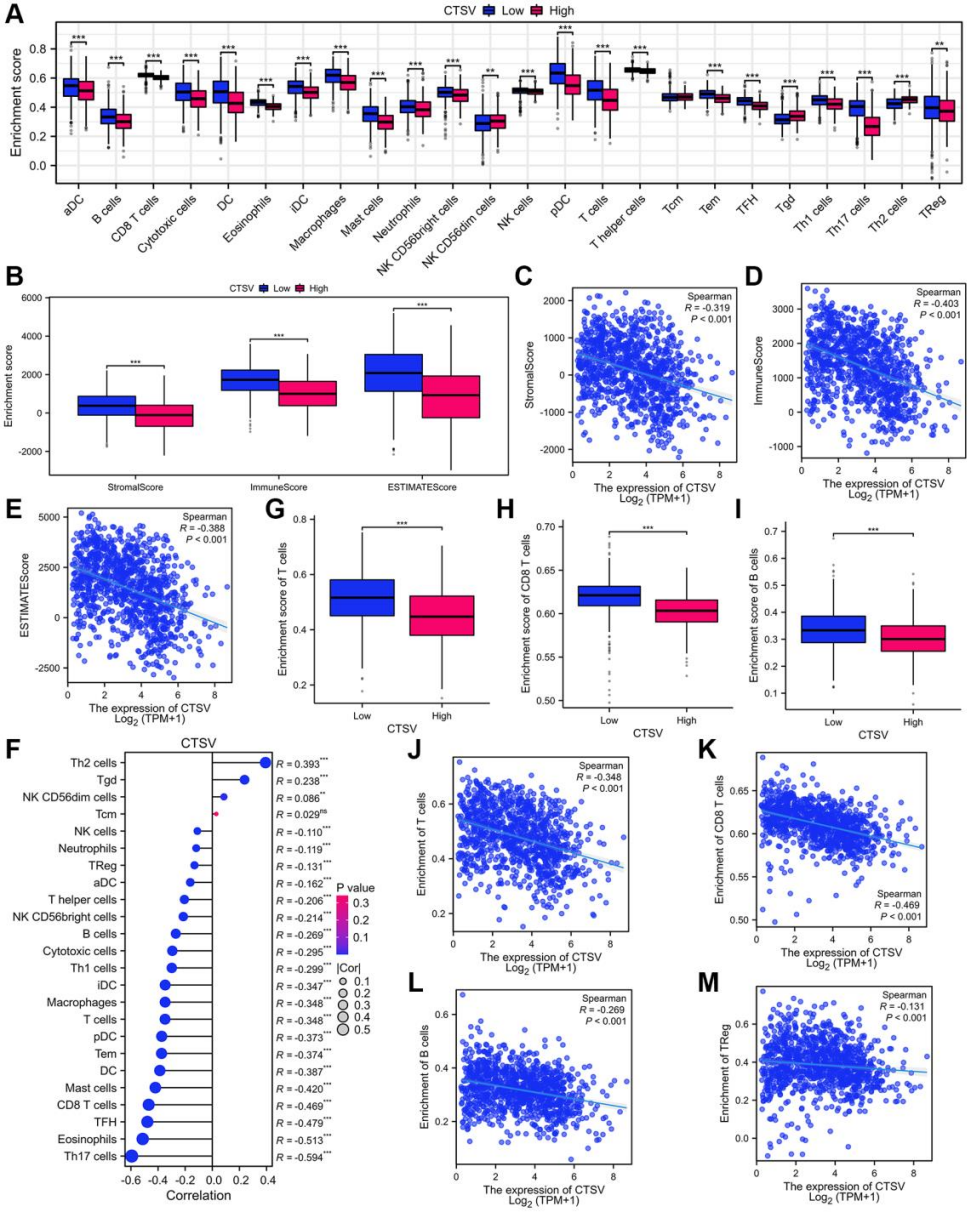
**Analysis of immune infiltration and lung cancer microenvironment based on CTSV expression in TCGA database.** (**A**) The immune cell infiltration analysis of lung cancer based on CTSV expression. (**B**–**E**) The expression of CTSV is correlated with significant immunosuppressive. (**F**–**M**) The expression of CTSV is negatively correlated with T cells (**G**, **J**), CD8 T cells (**H**, **K**), B cells (**I**, **L**), and TReg cells (**M**). ^*^P < 0.05; ^**^P < 0.01; ^***^P < 0.001.

We analyzed the TIMER database to determine the relationship between CTSV and infiltrated immune cells. Infiltration levels of immune cells are associated with upregulated gene copy numbers of CTSV (Figure 7A). Moreover, the high CTSV expression group showed lower levels of the StromalScore (Spearman R = −0.319), ImmuneScore (Spearman R = −0.403), and EstimateScore (Spearman R = −0.388) than that when compared with low expression CTSV group in lung cancer (Figure 7B–7E). Next, we further explored how CTSV affects the immune microenvironment in lung cancer. The lollipop chart showed that the high CTSV (TPM) expression level is negatively correlated with the infiltration level of most immune cells (Figure 7F). The correlation of infiltration level of immune cells and CTSV expression level (TPM) was accessed by Spearman analysis. Among the 24 subtypes of immune cells, especially T cells (R = −0.348, *P* < 0.001), CD8^+^T cells (R = −0.469, *P* < 0.001), B cells (R = −0.269, *P* < 0.001), and Treg cells (R = −0.131, *P* < 0.001) are negatively correlated with the high expression level of CTSV. (Figure 7G–7M). Our collective findings indicated that CTSV is essential to the immunosuppressive microenvironment of lung cancer.

### Knockdown of CTSV promotes T cell activity *in vitro*

**Figure 8:**
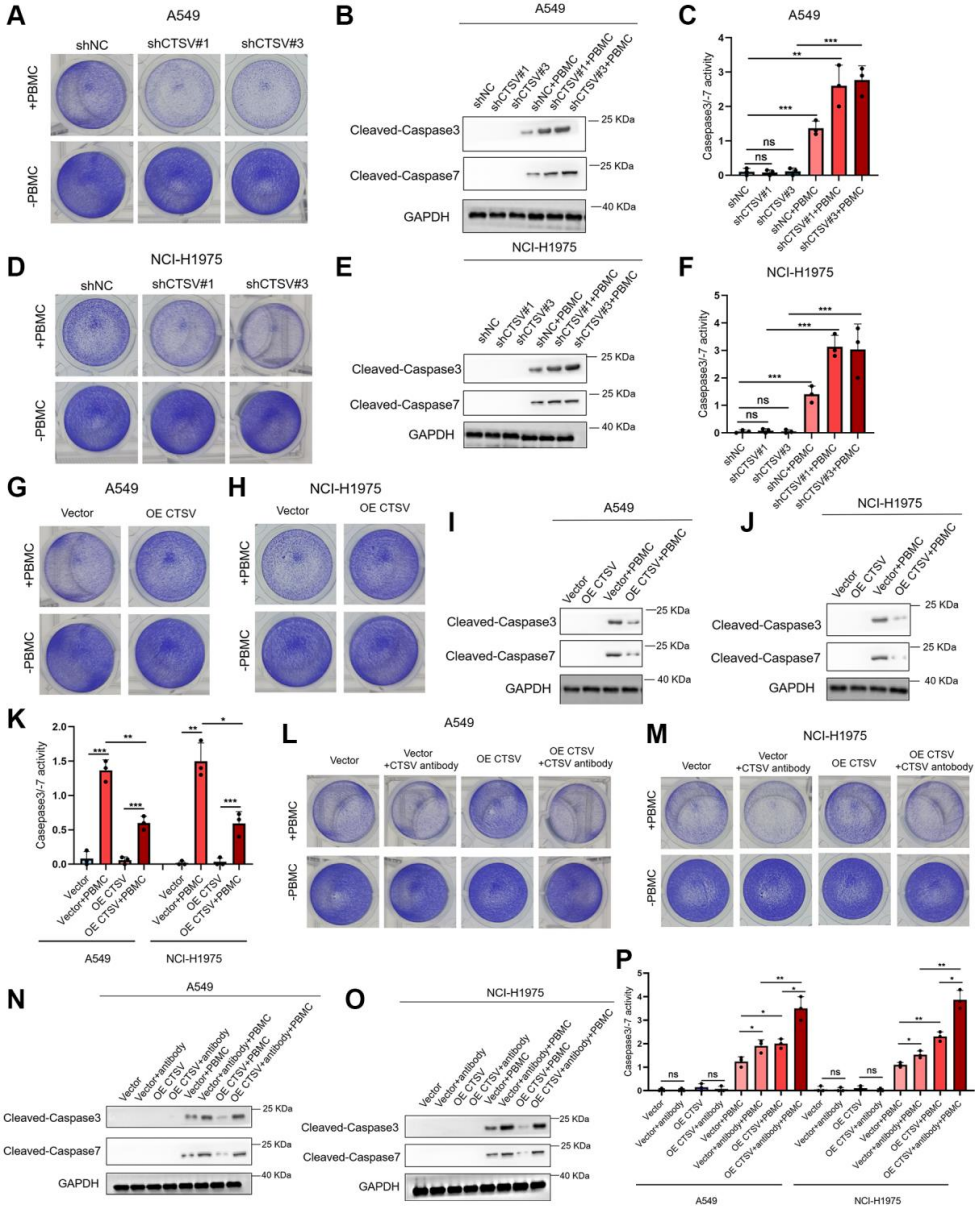
**CTSV inhibits T cell activity *in vitro*.** (**A**–**K**) Co-cultured the indicated stable A549 and NCI-H1975 cells with PBMCs for 48 h, then the remaining cells were fixed and dyed with 1% crystal violet. Western Blotting analysis of the remaining A549 and NCI-H1975 cells using anti-cleaved casepase3/−7 antibodies (**B**, **E**, **I**, **J**). Caspase 3/7 activity was measured using the Caspase-Glo 3/7 assay. Each bar represents the mean ± SEM (**C**, **F**, **K**). (**L**, **M**) The indicated stable A549 and NCI-H1975 cells were pre-treated with CTSV antibodies and then co-cultured with PBMCs for 48 h. Next, the remaining cells were fixed and dyed with 1% crystal violet. (**N**, **O**) Western Blotting analysis of the remaining A549 and NCI-H1975 cells from (**L**, **M**) using anti-cleaved casepase3/−7 antibody. (**P**) Caspase 3/−7 activity was measured using the Caspase-Glo 3/7 assay. ^**^P-value < 0.01 and ^***^P-value < 0.001.

We conducted co-culture experiments using lung cancer cells to examine the impact of CTSV depletion on T cell responses to identify whether CTSV regulates the activity of T cells. Depletion of CTSV enhanced T cell activity and contributed to the anti-tumor impact, as demonstrated by co-culturing A549 and NCI-H1975 cells with PBMCs (Figure 8A, 8D). Next, we further explored whether T cells mediate the apoptosis of cancer cells. Indeed, we observed increased caspase 3/7 cleavage and caspase 3/7 activity in A549 and NCI-H1975 cells when co-cultured with PBMCs (Figure 8B, 8C, 8E, 8F). In the co-culture assay, CTSV overexpression consistently reduces caspase 3/7 cleavage and caspase 3/7 activity in A549 and NCI-H1975 cells while favoring tumor immune escape (Figure 8G–8K). We treated cancer cells with CTSV antibodies and co-cultured them with PBMCs in light of our obvious findings that CTSV antibodies suppressed lung cancer cell migration and invasion. The results showed that CTSV blockage promoted T cell activity and increased caspase 3/−7 cleavage and activity in A549 and NCI-H1975 cells (Figure 8L–8P). Our research showed that CTSV suppressed T cell activity and encouraged immunological escape from the tumor. Hence, CTSV blockade increased T-cell activity and improved lung cancer cell apoptosis.

### Blockade of CTSV mitigates tumor metastasis *in vivo*

**Figure 9:**
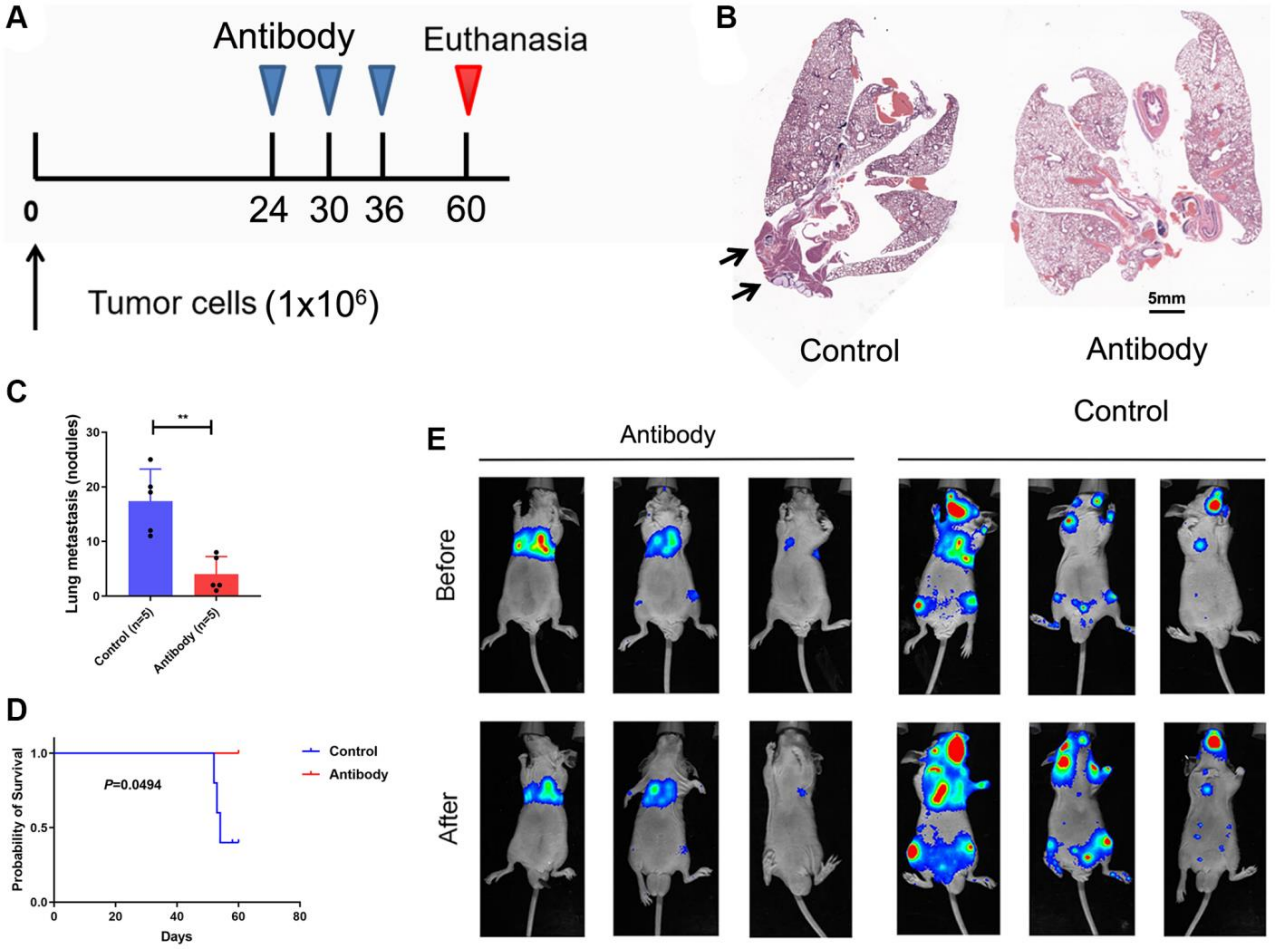
**Blockade CTSV suppresses tumor metastases in lung cancer.** (**A**–**C**) Lung metastasis nodules after tail vein injection of A549 cells (*n* = 3 mice per group). (**D**) Kaplan-Meier analysis of the indicated mice. (**E**) Intravenous injection of A549 cells for metastasis analysis. Representative images of antibody-treated mice and bioluminescent imaging (BLI) are shown (*n* = 3 mice per group). The representative data are repeated for three independent biological experiments. Student’s *t*-tests were used for data analysis; ^****^P-value < 0.0001.

We carried out tests using the lung cancer tail-vein-injection metastatic model to examine the *in vivo* anticancer effect of CTSV blocking (Figure 9A). As anticipated, CTSV inhibition greatly reduced lung metastases, increasing these mice’s survival times (Figure 9B–9D). Consistently, using A549 cells expressing firefly luciferase (A549-Luc cells) and stably expressing CTSV, as illustrated in Figure 6A, blockade of CTSV significantly suppressed lung metastases in a mouse model (Figure 9E). Our earlier findings suggest that CTSV blocking may be a useful treatment option for a minority of lung cancer patients with metastases.

## DISCUSSION

Due to its low survival rate, lung cancer continues to be the primary cause of cancer-related fatalities worldwide [29]. Therefore, it is crucial to research cutting-edge therapies and stop their recurrence and metastasis. One of the first indicators of cancer, the surrounding microenvironment, has been implicated in carcinogenesis, tumor maintenance, growth, advancement, and the interaction between tumor cells [5]. The tumor microenvironment underwent significant modifications early in carcinogenesis [2, 5]. The signal loops of paracrine mediate interactions between epithelial cells and their surroundings. These interactions result in the stability of the immunological microenvironment, remodeling of the extracellular matrix, and proliferation, progression, and invasion of tumors [11, 30, 31]. Combining the elements of the lung cancer cells’ microenvironment is necessary to comprehend biology and behavior. According to earlier research, lung cancer is associated with an elevated CTSV biomarker. It is still unknown how the dysregulation of CTSV in lung cancer functions and how it works.

Cathepsin can be directly secreted into the extracellular space to enter it [5]. Cysteine cathepsins’ varied location and distinct expression suggest that they have a more specialized function [6, 32]. According to reports, several immune cells constitute a significant source of extracellular cysteine cathepsins in inflammation, particularly in the brain [33, 34]. Recent research has demonstrated that active cathepsin can be found in the cytoplasm, nucleus, and plasma membrane of cells, which may contribute to neurodegeneration [35]. Cathepsins including cathepsins S, K, and V, which have potent elastolytic and collagenolytic activities, are primarily in charge of remodeling the extracellular matrix (ECM), after which they regulate bone remodeling and hormone production and contribute to several diseases [5, 36, 37]. Elastins, collagen, laminin, and proteoglycans are among the proteins that make up the extracellular matrix and are broken by cathepsins [5, 38, 39]. To gain a better understanding of how CTSV affects lung cancer metastasis and how protease dysregulation interferes with the homeostasis of healthy tissue. We measured the fibronectin, E-cadherin, and N-cadherin protein levels and discovered that CTSV downregulated these extracellular matrix proteins.

The catabolism of proteins and their degradation into amino acid components is the primary function of the proteases found within the acidic lumen of lysosomes. By cleaving adhesion molecules like fibronectin, E-cadherin, and N-cadherin, CTSV encouraged the spread of lung cancer, which was further supported by protein mass spectrometry and immunoprecipitation analysis. We reasoned that CTSV acts by being secreted from cells into the extracellular space, although these adhesion molecules are transmembrane or extracellular proteins or data offer a mechanistic understanding of this metastasis-related problem.

## CONCLUSION

**Figure 10:**
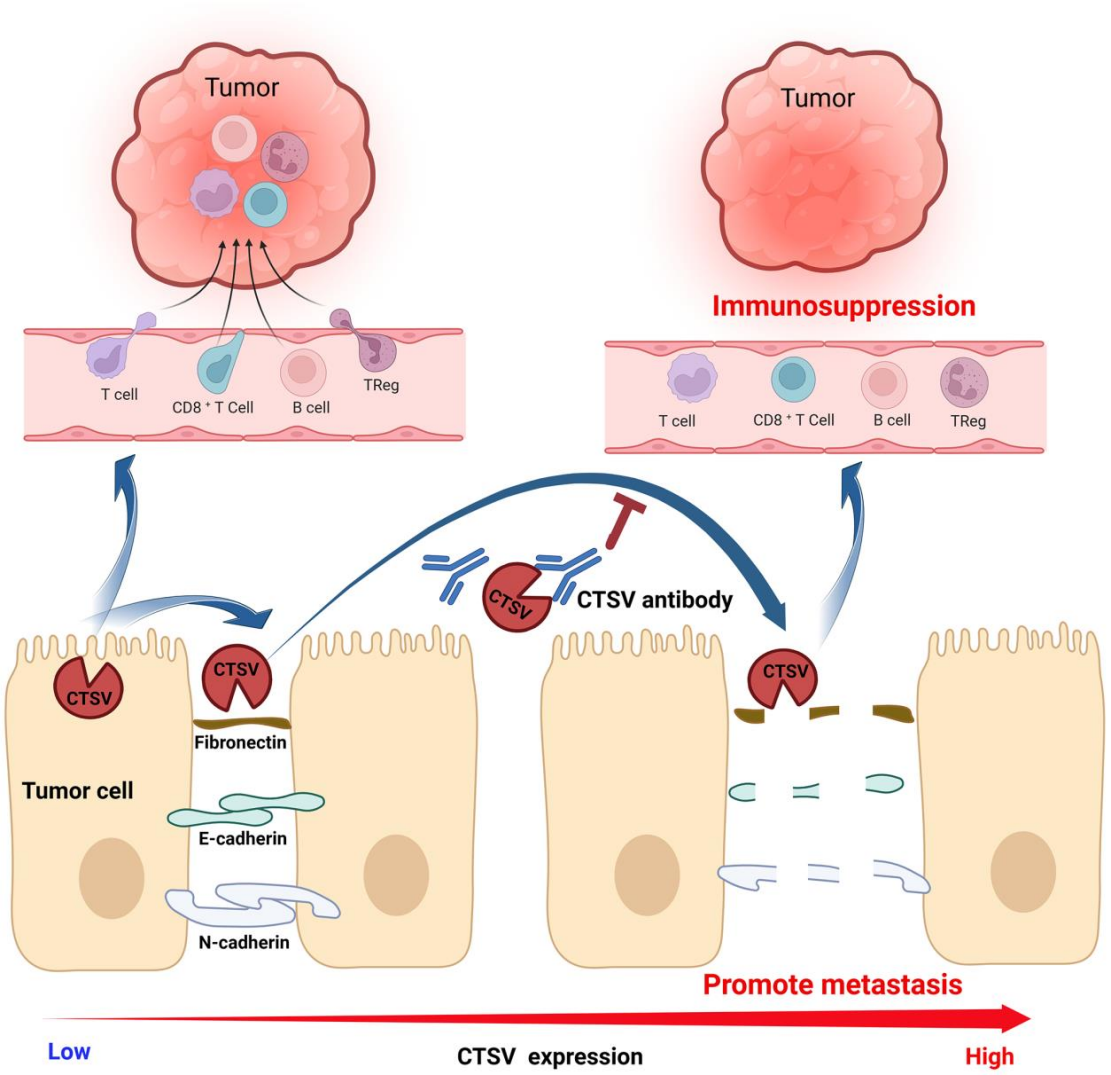
Graphic abstract A proposed model for the mechanism of CTSV in promoting lung cancer progression.

Our findings show lung cancer patients have higher CTSV, associated with a bad prognosis. In addition, we discovered that CTSV greatly speeds up lung cancer spread by cleaving off adhesion molecules like fibronectin, E-cadherin, and N-cadherin. Here, we created an antibody that specifically targets CTSV, which inhibited lung cancer cells’ adhesion, migration, and invasion by cleaving fibronectin, E-cadherin, and N-cadherinMore critically, overexpression of CTSV has been shown to suppress T cell function in lung cancer and is adversely correlated with immune cells penetrating tumors. We also demonstrated that CTSV inhibition reduces lung cancer metastasis *in vivo*. Targeting CTSV offers a cutting-edge therapeutic approach to halt lung cancer spread (Figure 10).

## Supplementary Materials

Supplementary Figure 1

## References

[1] Turk B, Turk D, Turk V. Protease signalling: the cutting edge. EMBO J. 2012; 31:1630–43. 10.1038/emboj.2012.42 22367392 PMC3321211

[2] Olson OC, Joyce JA. Cysteine cathepsin proteases: regulators of cancer progression and therapeutic response. Nat Rev Cancer. 2015; 15:712–29. 10.1038/nrc4027 26597527

[3] Devanathan G, Turnbull JL, Ziomek E, Purisima EO, Ménard R, Sulea T. Carboxy-monopeptidase substrate specificity of human cathepsin X. Biochem Biophys Res Commun. 2005; 329:445–52. 10.1016/j.bbrc.2005.01.150 15737607

[4] Nägler DK, Zhang R, Tam W, Sulea T, Purisima EO, Ménard R. Human cathepsin X: A cysteine protease with unique carboxypeptidase activity. Biochemistry. 1999; 38:12648–54. 10.1021/bi991371z 10504234

[5] Vizovišek M, Fonović M, Turk B. Cysteine cathepsins in extracellular matrix remodeling: Extracellular matrix degradation and beyond. Matrix Biol. 2019; 75-76:141–59. 10.1016/j.matbio.2018.01.024 29409929

[6] Sobotič B, Vizovišek M, Vidmar R, Van Damme P, Gocheva V, Joyce JA, Gevaert K, Turk V, Turk B, Fonović M. Proteomic Identification of Cysteine Cathepsin Substrates Shed from the Surface of Cancer Cells. Mol Cell Proteomics. 2015; 14:2213–28. 10.1074/mcp.M114.044628 26081835 PMC4528248

[7] Turk V, Stoka V, Vasiljeva O, Renko M, Sun T, Turk B, Turk D. Cysteine cathepsins: from structure, function and regulation to new frontiers. Biochim Biophys Acta. 2012; 1824:68–88. 10.1016/j.bbapap.2011.10.002 22024571 PMC7105208

[8] Quesada V, Ordóñez GR, Sánchez LM, Puente XS, López-Otín C. The Degradome database: mammalian proteases and diseases of proteolysis. Nucleic Acids Res. 2009; 37:D239–43. 10.1093/nar/gkn570 18776217 PMC2686449

[9] Biasizzo M, Javoršek U, Vidak E, Zarić M, Turk B. Cysteine cathepsins: A long and winding road towards clinics. Mol Aspects Med. 2022; 88:101150. 10.1016/j.mam.2022.101150 36283280

[10] Vasiljeva O, Reinheckel T, Peters C, Turk D, Turk V, Turk B. Emerging roles of cysteine cathepsins in disease and their potential as drug targets. Curr Pharm Des. 2007; 13:387–403. 10.2174/138161207780162962 17311556

[11] Bird PI, Trapani JA, Villadangos JA. Endolysosomal proteases and their inhibitors in immunity. Nat Rev Immunol. 2009; 9:871–82. 10.1038/nri2671 19935806

[12] Turk B. Targeting proteases: successes, failures and future prospects. Nat Rev Drug Discov. 2006; 5:785–99. 10.1038/nrd2092 16955069

[13] Vizin T, Christensen IJ, Nielsen HJ, Kos J. Cathepsin X in serum from patients with colorectal cancer: relation to prognosis. Radiol Oncol. 2012; 46:207–12. 10.2478/v10019-012-0040-0 23077459 PMC3472949

[14] Vižin T, Christensen IJ, Wilhelmsen M, Nielsen HJ, Kos J. Prognostic and predictive value of cathepsin X in serum from colorectal cancer patients. BMC Cancer. 2014; 14:259. 10.1186/1471-2407-14-259 24725597 PMC4021260

[15] Pan B, Yue Y, Ding W, Sun L, Xu M, Wang S. A novel prognostic signatures based on metastasis- and immune-related gene pairs for colorectal cancer. Front Immunol. 2023; 14:1161382. 10.3389/fimmu.2023.1161382 37180113 PMC10169605

[16] Zhang W, Wang S, Wang Q, Yang Z, Pan Z, Li L. Overexpression of cysteine cathepsin L is a marker of invasion and metastasis in ovarian cancer. Oncol Rep. 2014; 31:1334–42. 10.3892/or.2014.2967 24402045

[17] Jobs E, Ingelsson E, Risérus U, Nerpin E, Jobs M, Sundström J, Basu S, Larsson A, Lind L, Ärnlöv J. Association between serum cathepsin S and mortality in older adults. JAMA. 2011; 306:1113–21. 10.1001/jama.2011.1246 21878432

[18] Mai J, Finley RL Jr, Waisman DM, Sloane BF. Human procathepsin B interacts with the annexin II tetramer on the surface of tumor cells. J Biol Chem. 2000; 275:12806–12. 10.1074/jbc.275.17.12806 10777578

[19] Xiao Y, Cong M, Li J, He D, Wu Q, Tian P, Wang Y, Yang S, Liang C, Liang Y, Wen J, Liu Y, Luo W, et al. Cathepsin C promotes breast cancer lung metastasis by modulating neutrophil infiltration and neutrophil extracellular trap formation. Cancer Cell. 2021; 39:423–37.e7. 10.1016/j.ccell.2020.12.012 33450198

[20] Kim S, Lee KH, Choi HJ, Kim E, Kang S, Han M, Jeon HJ, Yun MY, Song GY, Lee HJ. Hederacolchiside A1 Suppresses Autophagy by Inhibiting Cathepsin C and Reduces the Growth of Colon Cancer. Cancers (Basel). 2023; 15:1272. 10.3390/cancers15041272 36831614 PMC9953978

[21] Schurigt U, Eilenstein R, Gajda M, Leipner C, Sevenich L, Reinheckel T, Peters C, Wiederanders B, Bräuer R. Decreased arthritis severity in cathepsin L-deficient mice is attributed to an impaired T helper cell compartment. Inflamm Res. 2012; 61:1021–9. 10.1007/s00011-012-0495-x 22674323

[22] Sereesongsaeng N, McDowell SH, Burrows JF, Scott CJ, Burden RE. Cathepsin V suppresses GATA3 protein expression in luminal A breast cancer. Breast Cancer Res. 2020; 22:139. 10.1186/s13058-020-01376-6 33298139 PMC7726886

[23] Leng Y, Chen R, Chen R, He S, Shi X, Zhou X, Zhang Z, Chen AF. HMGB1 mediates homocysteine-induced endothelial cells pyroptosis via cathepsin V-dependent pathway. Biochem Biophys Res Commun. 2020; 532:640–6. 10.1016/j.bbrc.2020.08.091 32912629

[24] Niwa Y, Suzuki T, Dohmae N, Umezawa K, Simizu S. Determination of cathepsin V activity and intracellular trafficking by N-glycosylation. FEBS Lett. 2012; 586:3601–7. 10.1016/j.febslet.2012.08.001 22967898

[25] Yang L, Zeng Q, Deng Y, Qiu Y, Yao W, Liao Y. Glycosylated Cathepsin V Serves as a Prognostic Marker in Lung Cancer. Front Oncol. 2022; 12:876245. 10.3389/fonc.2022.876245 35494076 PMC9043764

[26] Akkari L, Gocheva V, Kester JC, Hunter KE, Quick ML, Sevenich L, Wang HW, Peters C, Tang LH, Klimstra DS, Reinheckel T, Joyce JA. Distinct functions of macrophage-derived and cancer cell-derived cathepsin Z combine to promote tumor malignancy via interactions with the extracellular matrix. Genes Dev. 2014; 28:2134–50. 10.1101/gad.249599.114 25274726 PMC4180975

[27] Joyce JA, Pollard JW. Microenvironmental regulation of metastasis. Nat Rev Cancer. 2009; 9:239–52. 10.1038/nrc2618 19279573 PMC3251309

[28] Mason SD, Joyce JA. Proteolytic networks in cancer. Trends Cell Biol. 2011; 21:228–37. 10.1016/j.tcb.2010.12.002 21232958 PMC3840715

[29] Sung H, Ferlay J, Siegel RL, Laversanne M, Soerjomataram I, Jemal A, Bray F. Global Cancer Statistics 2020: GLOBOCAN Estimates of Incidence and Mortality Worldwide for 36 Cancers in 185 Countries. CA Cancer J Clin. 2021; 71:209–49. 10.3322/caac.21660 33538338

[30] Dennemärker J, Lohmüller T, Mayerle J, Tacke M, Lerch MM, Coussens LM, Peters C, Reinheckel T. Deficiency for the cysteine protease cathepsin L promotes tumor progression in mouse epidermis. Oncogene. 2010; 29:1611–21. 10.1038/onc.2009.466 20023699 PMC3082505

[31] Guinec N, Dalet-Fumeron V, Pagano M. "In vitro" study of basement membrane degradation by the cysteine proteinases, cathepsins B, B-like and L. Digestion of collagen IV, laminin, fibronectin, and release of gelatinase activities from basement membrane fibronectin. Biol Chem Hoppe Seyler. 1993; 374:1135–46. 10.1515/bchm3.1993.374.7-12.1135 8129860

[32] Mohamed MM, Sloane BF. Cysteine cathepsins: multifunctional enzymes in cancer. Nat Rev Cancer. 2006; 6:764–75. 10.1038/nrc1949 16990854

[33] Hayashi Y, Koyanagi S, Kusunose N, Okada R, Wu Z, Tozaki-Saitoh H, Ukai K, Kohsaka S, Inoue K, Ohdo S, Nakanishi H. The intrinsic microglial molecular clock controls synaptic strength via the circadian expression of cathepsin S. Sci Rep. 2013; 3:2744. 10.1038/srep02744 24067868 PMC3783043

[34] Wendt W, Lübbert H, Stichel CC. Upregulation of cathepsin S in the aging and pathological nervous system of mice. Brain Res. 2008; 1232:7–20. 10.1016/j.brainres.2008.07.067 18694734

[35] Bonneh-Barkay D, Wiley CA. Brain extracellular matrix in neurodegeneration. Brain Pathol. 2009; 19:573–85. 10.1111/j.1750-3639.2008.00195.x 18662234 PMC2742568

[36] Brix K, Dunkhorst A, Mayer K, Jordans S. Cysteine cathepsins: cellular roadmap to different functions. Biochimie. 2008; 90:194–207. 10.1016/j.biochi.2007.07.024 17825974

[37] Kong QF, Sun B, Bai SS, Zhai DX, Wang GY, Liu YM, Zhang SJ, Li R, Zhao W, Sun YY, Li N, Wang Q, Peng HS, et al. Administration of bone marrow stromal cells ameliorates experimental autoimmune myasthenia gravis by altering the balance of Th1/Th2/Th17/Treg cell subsets through the secretion of TGF-beta. J Neuroimmunol. 2009; 207:83–91. 10.1016/j.jneuroim.2008.12.005 19174310

[38] Langley SR, Willeit K, Didangelos A, Matic LP, Skroblin P, Barallobre-Barreiro J, Lengquist M, Rungger G, Kapustin A, Kedenko L, Molenaar C, Lu R, Barwari T, et al. Extracellular matrix proteomics identifies molecular signature of symptomatic carotid plaques. J Clin Invest. 2017; 127:1546–60. 10.1172/JCI86924 28319050 PMC5373893

[39] Mihalko EP, Brown AC. Material Strategies for Modulating Epithelial to Mesenchymal Transitions. ACS Biomater Sci Eng. 2018; 4:1149–61. 10.1021/acsbiomaterials.6b00751 33418653 PMC13005913

