# Supplementary material for: Cathepsin V drives lung cancer progression by shaping the immunosuppressive environment and adhesion molecules cleavage
Source: Aging (Albany NY). 2023 Dec 8;15(23):13961–79. doi: 10.18632/aging.205278 (PMC10756122; doi:10.18632/aging.205278)
Supplement: Supplementary Figure 1 [file aging-15-205278-s001.pdf]

SUPPLEMENTARY FIGURE

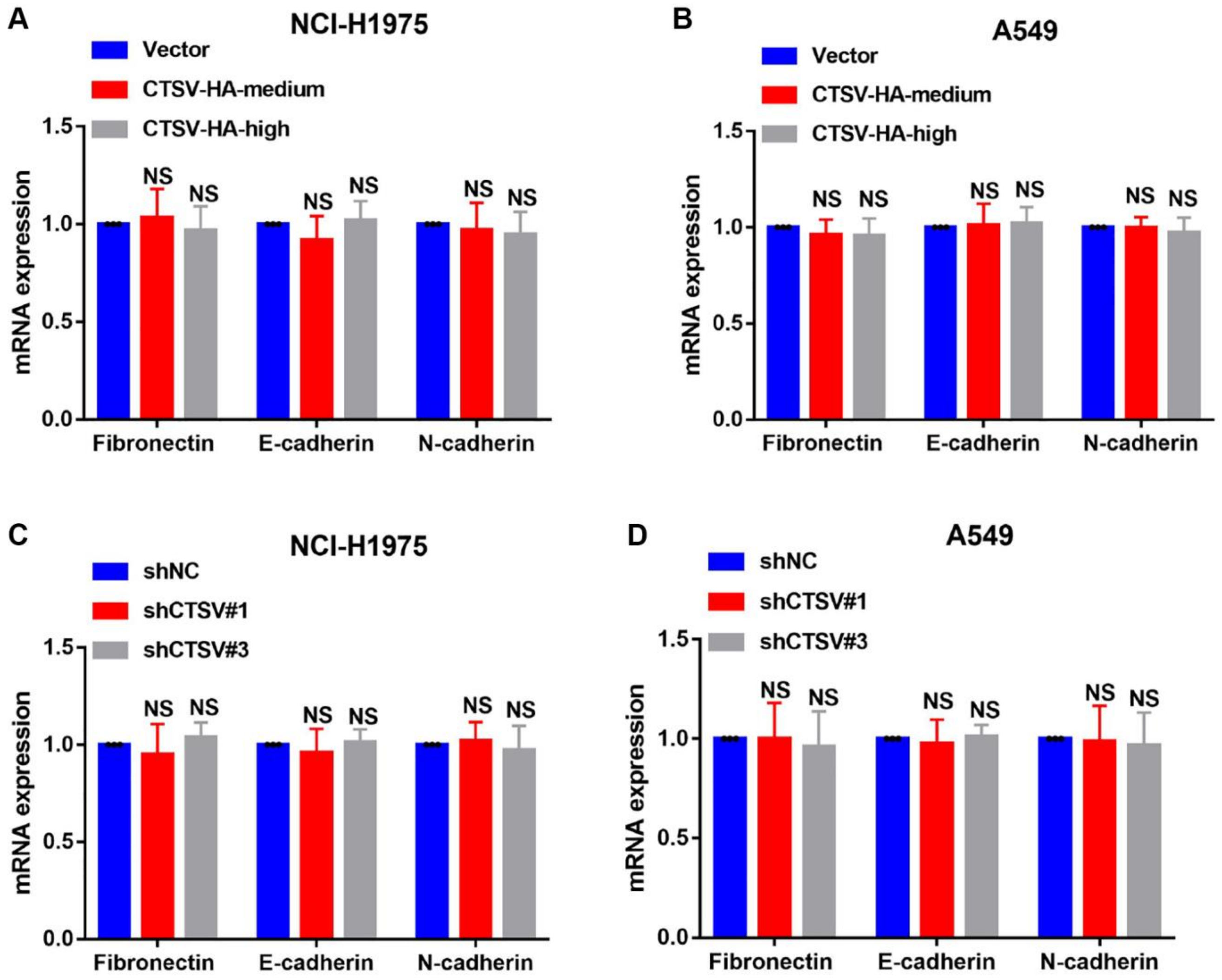

Supplementary Figure 1. CTSV has no effect on fibronectin, E-caderin and N-cadherin at transcriptional level. (**A**–**D**) The relative mRNA levels of fibronectin, N-cadherin and E-cadherin were normalized to the GAPDH level in the indicated stable cells as determined by qRT-PCR. The results are expressed as the mean ± SD of three independent experiments. Abbreviation: NS: no significant difference.
